# Virulence Gene Profiles of Extended-Spectrum β-Lactamase (ESBL)-Producing *Escherichia coli* Isolated from Turkeys in Hungary: A Whole-Genome Sequencing Study

**DOI:** 10.3390/vetsci12121141

**Published:** 2025-11-29

**Authors:** Ádám Kerek, Ábel Szabó, Gergely Tornyos, Eszter Kaszab, Krisztina Bali, Ákos Jerzsele

**Affiliations:** 1 Department of Pharmacology and Toxicology, University of Veterinary Medicine Budapest, H-1078 Budapest, Hungary; szabo.abel@student.univet.hu (Á.S.); tornyos.gergely@student.univet.hu (G.T.); jerzsele.akos@univet.hu (Á.J.); 2 National Laboratory of Infectious Animal Diseases, Antimicrobial Resistance, Veterinary Public Health and Food Chain Safety, University of Veterinary Medicine Budapest, H-1078 Budapest, Hungary; 3 One Health Institute, University of Debrecen, Nagyerdei krt. 98, H-4032 Debrecen, Hungary; 4 Department of Microbiology and Infectious Diseases, University of Veterinary Medicine, István u 2, H-1078 Budapest, Hungary

**Keywords:** *Escherichia coli*, virulence factors, NGS, AMR, turkeys

## Abstract

**Background:** *Escherichia coli* strains associated with poultry are increasingly recognized as reservoirs of both virulence and resistance genes, posing significant zoonotic risks throughout the food production chain. However, the genotypic architecture and pathogenic potential of isolates from large-scale turkey farms remain under characterized, particularly in the context of extended-spectrum β-lactamase (ESBL) production. **Methods:** A total of 160 ESBL-producing *E. coli* isolates were collected from healthy turkeys on intensive Hungarian farms. Whole genome sequencing (WGS) was performed to characterize virulence factors. Functional annotation included screening for fimbrial adhesins, iron acquisition systems, secretion pathways, and autotransporter toxins, using VirulenceFinder and Prodigal-based genome annotations. Data analysis included assembly quality control with QUAST and BUSCO, and comprehensive virulome profiling. **Results:** The isolates exhibited a functionally diverse virulence profile encompassing classical ExPEC-associated colonization factors (type I, P, S fimbriae; curli; ECP), multiple iron acquisition systems (enterobactin, salmochelin, aerobactin, yersiniabactin, and heme uptake), and key secretion systems (LEE-associated T3SS and T2SS). Genetic hallmarks of avian pathogenic *E. coli* (APEC), uropathogenic pathogenic *E. coli* (UPEC), and enteropathogenic *E. coli* (EPEC) pathotypes co-occurred in 44% of the isolates, indicating a mosaic virulence landscape. Notably, serine protease autotransporters of Enterobacteriaceae (SPATE) toxins (Vat, Pic) and ColV-type plasmid-associated modules were frequently detected. All isolates were confirmed by ESBL producers, highlighting their antimicrobial resistance potential. **Conclusions:** This study reveals that *E. coli* strains isolated from turkeys possess a complex, host-adapted virulence repertoire capable of supporting both enteric and extraintestinal infections. The co-occurence of APEC-, UPEC-, and EPEC-like traits—combined with ESBL production—underscores their One Health relevance. These findings support the need for host-specific surveillance, functional validation, and integrative control strategies in poultry systems.

## Simple Summary

*Escherichia coli* is a common bacterium found in both humans and animals, but some strains can carry genes that make them pathogenic or resistant to antibiotics. In poultry, these bacteria may act as hidden reservoirs of disease-causing and drug-resistant traits, which can potentially affect animal and human health. In this study, we collected and analyzed 160 *E. coli* strains from healthy turkeys raised on intensive farms in Hungary. All strains produced extended-spectrum β-lactamase (ESBL), an enzyme that makes them resistant to many antibiotics. Using whole-genome sequencing, we examined which virulence genes these bacteria carried. The results showed a complex combination of genetic features that help *E. coli* stick to host tissues, capture iron, and release toxins—traits usually linked to strains that cause urinary or intestinal infections in humans and birds. Many isolates also carried mobile genetic elements like ColV plasmids, which can spread these traits to other bacteria. These findings suggest that turkeys may carry *E. coli* strains with the potential to cause disease and resist treatment, even when the animals appear healthy. This highlights the importance of monitoring such bacteria as part of a One Health approach to food safety and antimicrobial resistance.

## 1. Introduction

*Escherichia coli* is a facultative anaerobic, Gram-negative bacterium that is widely distributed in the intestinal tract of warm-blooded animals and serves as a key indicator organism in environmental and food safety assessments [1]. While most *E. coli* strains are harmless commensals, certain genotypes—through the acquisition of various virulence factors—can cause disease. In poultry production, particular attention is given to avian pathogenic *E. coli* (APEC) strains [2,3].

Colibacillosis caused by APEC strains leads to significant economic losses in the global poultry industry, including turkey farming [4]. These strains harbor a wide range of virulence-associated genes, such as adhesins (*fimH*, *papC*), invasion and immune evasion factors (*iss*, *ompT*), and iron acquisition systems (*iutA*, *iroN*). These determinants enable the bacteria to traverse the intestinal mucosa, colonize the respiratory tract, and establish extraintestinal infections [5]. Many of these virulence genes are plasmid-encoded, facilitating horizontal gene transfer and contributing to genetic plasticity and adaptive potential [6].

In parallel with virulence, antimicrobial resistance in *E. coli* has become an increasing global concern. Over recent decades, extended-spectrum β-lactamase (ESBL)-producing strains have emerged and spread worldwide [7]. These strains are capable of hydrolyzing third-generation cephalosporins and other β-lactam antibiotics, complicating treatment options [8]. Alarmingly, ESBL-producing *E. coli* often co-harbor virulence factors on the same plasmids, further amplifying their clinical significance [9,10]. Accordingly, it is essential to assess the prevalence and virulence gene content of these strains in food-producing animals, such as turkeys, which may serve as reservoirs for zoonotic pathogens [11].

Phylogenetic classification of *E. coli* into groups such as A, B1, B2, and D enables estimation of their pathogenic potential. Extraintestinal pathogenic strains typically belong to groups B2 and D, while commensal strains are more frequently found in groups A and B1 [12]. However, the phylogenetic distribution and virulence gene profiles of *E. coli* from poultry may vary depending on species, husbandry practices, and geographic region [13,14].

In Hungary, turkey production represents the second-largest poultry sector after broiler chickens, there is limited genotypic data on *E. coli* strains isolated from turkeys, particularly regarding the co-occurrence of virulence factors and ESBL production [15,16]. This gap underscores the need for targeted investigations, especially in the context of zoonotic risk and the dissemination of resistance genes, aligned with the One Health framework [17]. Preserving antibiotic efficacy for future generations is a collective responsibility, and it hinges on responsible, targeted antimicrobial use and reduced application frequency [18,19,20]. Achieving these goals requires adherence to effective vaccination and disinfection protocols [21] and—where feasible—the implementation of alternative strategies such as probiotics [22], antimicrobial peptides [23], and plant-derived essential oils and extracts [24,25,26,27,28]. Research into such alternatives is rapidly expanding, with an increasing number of natural compounds being explored as potential antibiotic substitutes [29,30,31].

The aim of this study was to investigate the molecular virulence gene profiles of *E. coli* strains isolated from large-scale turkey farms in Hungary. All strains were pre-screened and confirmed to be ESBL producers. The findings may contribute to improved risk assessment of *E. coli* in turkey flocks and support the development of targeted preventive and therapeutic strategies.

## 2. Materials and Methods

### 2.1. Sampling and Identification of Escherichia coli Strains

Between 2022 and 2023, trachea and cloaca specimens were collected from clinically healthy domestic turkeys kept under intensive farming conditions. At each site, 15 tracheal and 15 cloacal swabs were obtained using aluminum-handled Amies transport swabs without charcoal (Biolab Zrt., Budapest, Hungary). The samples were streaked onto ChromoBio^®^ Coliform agar (Biolab Zrt.) plates to isolate presumptive *E. coli* colonies. These were further subcultured onto tryptone soya agar and incubated at 41 °C for 18 to 24 h. Isolate identification was conducted using matrix-assisted laser desorption/ionization time-of-flight mass spectrometry (MALDI-TOF MS; Flextra-LAB Ltd., Budapest, Hungary) with Biotyper software (version 12.0, Bruker Daltonics, Bremen, Germany) [32]. Verified isolates were cryogenically preserved at –80 °C using the Microbank™ storage system (Pro-Lab Diagnostics, Richmond Hill, ON, Canada).

The *E. coli* isolates analyzed in this study were pre-selected based on prior phenotypic screening for multidrug resistance, including confirmed ESBL production. This preliminary testing was performed as part of a previously characterized in an independent study. Accordingly, only isolates (*n* = 160/470; 34%) with relevant ESBL resistance profiles were subjected to whole-genome sequencing in the present work, which focuses exclusively on the genotypic characterization of these pre-screened strains.

### 2.2. Whole-Genome Sequencing

To investigate the genetic determinants underlying ESBL production, total DNA was extracted using the Zymo Quick-DNA Fungal/Bacterial Miniprep Kit, in accordance with the manufacturer’s instructions (https://files.zymoresearch.com/protocols/_d6005_quick-dna_fungal-bacterial_miniprep_kit.pdf (accessed on 24 November 2025)). Cell disruption was performed to extract genomic DNA and achieved via mechanical lysis using the Qiagen TissueLyzer LT at 50 Hz for 5 min. The resulting lysates were stored at −20 °C until further processing. Although library preparation followed an Illumina-compatible workflow, the final sequencing was performed on the MGI DNBSEQ-G400RS platform after library conversion using the MGIEasy Universal Library Conversion Kit [33,34].

Library preparation was carried out with the Vazyme TruePrep DNA Library Prep Kit V2 (TD501-01) in combination with Nextera XT Index Kits (Set A and B). For each sample, 50 ng of genomic DNA was mixed with 5 µL tagmentation buffer, 2.5 µL transposase enzyme, and 25 µL nuclease-free water, followed by incubation at 55 °C for 10 min. DNA fragments were purified using the Geneaid Gel/PCR Extraction Kit.

PCR amplification of indexed sequencing libraries involved 5 µL amplification buffer, 2.5 µL primer mix, 0.5 µL polymerase, 2.5 µL of i5 and i7 indexing primers, and 12 µL of purified DNA. Thermocycling conditions were as follows: initial extension at 72 °C for 3 min, denaturation at 98 °C for 30 s, followed by 10 cycles of 98 °C for 15 s, 60 °C for 30 s, and 72 °C for 3 min, with a final elongation at 72 °C for 5 min. Post-amplification, the libraries were purified and quantified using the Qubit dsDNA HS Assay Kit.

For downstream sequencing on the MGI platform, libraries were adapted using the MGIEasy Universal Library Conversion Kit (App-A). After adjusting the volume to 22 µL, samples underwent adapter ligation using AC-PCR reagents, followed by PCR (initial denaturation at 98 °C for 3 min; 10 cycles at 98 °C for 30 s, 62 °C for 15 s, 72 °C for 30 s; final extension at 72 °C for 5 min). Cleanup was performed using magnetic beads, and concentrations were re-assessed.

Subsequently, single-stranded DNA was generated via thermal denaturation at 95 °C for 3 min and snap-cooled on ice. This was followed by splint ligation and enzymatic digestion, with appropriate buffer additions at each step. DNA was stabilized with Stop Buffer and quantified using the Qubit ssDNA HS Assay. DNA nanoball (DNB) generation followed, using App-C chemistry with a thermal cycling protocol including steps at 95 °C, 65 °C, 40 °C, and final incubation at 30 °C with enzyme mixes. The resulting DNBs were stabilized and stored at 4 °C.

Pooled libraries, adjusted to a maximum concentration of 20 ng/µL, were loaded onto a DNBSEQ-G400RS sequencing flow cell and sequenced using the HotMPS High Throughput Kit (FCL PE150).

### 2.3. Bioinformatic Analysis

To ensure the integrity and reliability of sequencing data, raw reads underwent initial quality assessment using FastQC (v0.11.9) [35], Fastp (v0.23.2-3) [36], and Bloocoo (v1.0.7) [37]. These tools enabled the detection of potential adapter contamination, base quality anomalies, and k-mer frequency deviations. TrimGalore (v0.6.6) [38] was applied to remove low-quality sequences and adaptor artifacts. High-quality reads were subsequently assembled into contigs using both MEGAHIT (v1.2.9) [39] and SPAdes (v4.0.0) [40], the resulting assemblies were then merged with GAM-NGS (v1.1b) [41] to enhance assembly continuity and accuracy.

Assembly evaluation was conducted using QUAST (v5.2) [42] to assess general metrics, while BUSCO (v5) [43] was used to determine completeness based on lineage-specific orthologs. GenomeScope (v2.2) [44] provided estimates of genome size, coverage depth, and k-mer distribution parameters. Prodigal (v2.6.3) [45] was utilized to predict coding sequences and open reading frames (ORFs).

Taxonomic confirmation was achieved through CheckM (v1.2.2) [46] and Kraken (v1.1.1) [47]. Chromosomal resistance determinants were identified using ResFinder (v4.1) [48,49,50], while genome-wide variant calling and SNP profiling were conducted with Snippy (v4.6.0) [51]. Serotyping was performed via Ectyper (v1.0) [52], and virulence gene profiling was completed using VirulenceFinder (v2.0) [49,53,54]. Pathotype associations of individual virulence genes were inferred based on their predominant linkage in the scientific literature to APEC, UPEC, or EPEC pathotypes [55,56].

Average nucleotide identity (ANI) calculations were carried out with ANI tool v2.0 [57], using *E. coli* strain SYNB8802 (RefSeq ID: GCF_020995495.1) as the closest reference genome available in the NCBI RefSeq database [58].

## 3. Results

### 3.1. Virulence Gene Repertoire Identified

A total of 160 *E. coli* strains isolated from turkeys were analyzed for virulence gene content. Altogether, 157 distinct virulence-associated genes were identified (Supplementary Table S1). Many of the detected genes are known to facilitate colonization and persistence within the host organism. Specifically, 51 unique colonization-related virulence genes were identified (Table 1).

The detected virulence factors included both fimbrial and non-fimbrial adhesion genes, which are essential for attachment to intestinal epithelial cells, biofilm formation, and evasion of host immune responses.

Genes involved in curli fiber biosynthesis (*csgB*, *csgF*, *csgG*) were present in several isolates (25%). These contribute to biofilm formation and environmental persistence and have been previously reported in both APEC and UPEC strains. Similarly, the outer membrane protein gene *ompA* (24%) was commonly detected, which may promote adhesion to host surfaces and has also been associated with APEC and UPEC pathotypes.

Among the fimbrial adhesion gene families, multiple components of the *fim* operon (*fimA–I*; 36%) were identified. This operon encodes type I fimbriae, which plays a central role in the pathogenicity of enteropathogenic (*EPEC*), enterohemorrhagic (*EHEC*), and UPEC strains. Of particular note, the *fimH* gene (29%)—encoding the terminal adhesin subunit of the fimbria—was frequently detected, as it mediates direct binding to host cells.

Genes from the *pap* (2%) operon (*papB–papK*), which encode the structural and regulatory elements of P fimbriae, were also found. These are primarily associated with UPEC strains and contribute to uroepithelial adhesion.

In addition, genes linked to S fimbriae (*sfaB–sfaY;* 2%), commonly found in UPEC strains, were detected. Genes involved in the biosynthesis of F4 (K88) fimbriae (*faeC–faeJ;* 4%)—typically associated with enterotoxigenic *E. coli* (ETEC)—were also present. The *fdeC* gene (15%), which facilitates binding to fibronectin, was identified as well and may contribute to adhesion to host extracellular matrix components.

Genes related to the *E. coli* common pilus (ECP), specifically those of the *yag/ecp* operon (17%), were also detected. This gene cluster is often found in EPEC strains and plays a general role in colonization and biofilm formation. Moreover, individual components of the *pil* operon (*pilG*, *pilH;* 6%) were present. These genes are involved in the biogenesis of type IV pili and are typically linked to EPEC strains.

The *E. coli* isolates from turkeys displayed a broad array of genes linked to bacterial fitness and persistence (Table 2). These genes are primarily involved in environmental adaptation, survival within the host, and acquisition of essential nutrients, particularly iron.

Among the most prominent were those associated with siderophore biosynthesis and transport. These included components of the *ent* operon (*entA–F*, *entS*) and the *fep* genes (*fepA–G*, *fes*) involved in the transport of enterobactin. Additionally, genes encoding components of the aerobactin system (*iucA–D*, *iutA*) and the yersiniabactin system (*irp1*, *irp2*, *ybtA–X*) were detected, which are typically associated with extraintestinal pathogenic *E. coli* (ExPEC) and APEC strains. The *iro* genes (*iroB–E*, *iroN*) encoding the salmochelin transporter were also present, further supporting iron acquisition under iron-limited conditions. Moreover, the *chu* operon (*chuA–Y*) was identified, enabling the utilization of heme as an iron source. Collectively, the diversity of iron acquisition systems reflects the adaptive capacity and pathogenic potential of these isolates.

The presence of *kps* genes (*kpsD*, *kpsM*, *kpsT*) indicates the capability for capsular polysaccharide biosynthesis and export, which contributes to resistance against complement-mediated lysis and helps evade host immune defenses. The *waaF* and *waaG* genes, involved in lipopolysaccharide (LPS) core synthesis, play a critical role in maintaining cell envelope integrity and mediating host–pathogen interactions.

A wide range of genes associated with flagellar structure and function was also detected, including *flgC–I*, *flhA*, *fliA–P*, *fleN*, and *fleQ*. These genes are essential for bacterial motility, which enhances colonization, biofilm formation, and tissue access within the host. The *motB* and *motC* motor protein genes, along with regulatory elements, are involved in fine-tuning flagellar-driven motility.

Genes of the *alg* operon (*alg8*, *algA*, *algB*, *algC*, *algI*, *algU*, *algW*), which govern alginate biosynthesis and its regulation, were also present. These genes likely contribute to environmental persistence and immune evasion within the host through biofilm formation. Furthermore, the *mucD* gene, encoding a serine protease, was detected; it is implicated in stress response and may also play a role in regulating virulence.

The *E. coli* isolates of turkey origin also harbored genes associated with toxin production and bacterial secretion systems, which play a fundamental role in pathogenicity and host–pathogen interactions (Table 3).

Among the identified genes, *pic* encodes a serine protease autotransporter implicated in immune evasion and enhanced intestinal colonization. This gene is most commonly found in enteroaggregative *E. coli* (EAEC) strains. Additionally, the *vat* gene was detected, which encodes a vacuolating autotransporter toxin. It is typically associated with the APEC pathotype and may contribute to tissue and cellular damage.

A diverse set of genes linked to the type III secretion system (T3SS) was identified, including *espL1*, *espL4*, *espR1–R4*, *espX1–X6*, and *espY1–Y4*. These genes are characteristically associated with EHEC and EPEC pathotypes and are located within the locus of enterocyte effacement (LEE) pathogenicity island. They are critical for bacterial adhesion to host epithelial cells, actin cytoskeleton rearrangement, and disruption of epithelial barrier integrity. The *espR1–R4* regulatory genes are involved in the fine-tuned expression of the LEE operon, while the *espX* and *espY* gene families encode effector proteins that modulate various host cellular processes.

Furthermore, genes encoding components of the type II secretion system (T2SS), such as *gspC*, *gspD*, and *gspE–M*, were also detected. These genes facilitate the secretion of proteins, including a range of extracellular enzymes and toxins. Several of these components (*gspE–M*) are associated with EHEC and ETEC pathotypes, while others (*gspC*, *gspD*) are more broadly distributed among Gram-negative bacteria. The presence of the T2SS suggests that the isolates may possess the capacity to secrete various virulence factors and extracellular proteins, thereby enhancing their colonization potential and infectivity.

### 3.2. Distribution of Virulence Genes Based on Pathotypes

**Figure 1:**
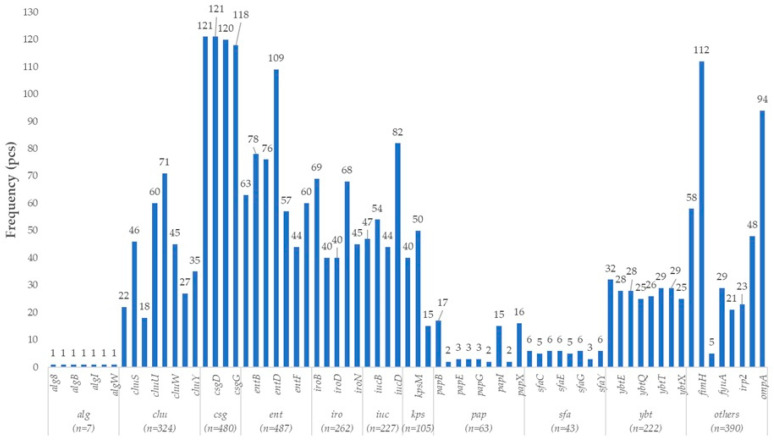
Prevalence of uropathogenic *Escherichia coli* (UPEC)-associated virulence genes in turkey-derived *Escherichia coli* isolates (*n* = 160).

The *E. coli* isolates of turkey origin exhibited a substantial number of genes typically associated with UPEC, forming a complex repertoire of colonization and virulence factors (Figure 1). These strains carried multiple genes involved in alginate biosynthesis (*alg8*, *algA*, *algB*, *algC*, *algI*, *algU*, *algW*), which are known to contribute to biofilm formation and enhance stress tolerance. In addition, curli biosynthesis genes (*csgB*, *csgD*, *csgF*, *csgG*) were also widely detected, supporting adhesion and stabilization of the biofilm matrix.

The isolates harbored a comprehensive and redundant set of iron acquisition systems, including the *chu* gene cluster (*chuA–Y*) enabling heme utilization, the enterobactin system (*entA–F*, *entS*), genes for aerobactin synthesis and transport (*iucA–D*, *iutA*), the yersiniabactin gene cluster (*irp1*, *irp2*, *ybtA–X*), and the salmochelin transporter genes (*iroB–E*, *iroN*). The co-occurrence of multiple, partially redundant siderophore systems suggests that these strains are well-equipped to survive under iron-limited conditions within the host.

With respect to colonization, fimbrial gene systems characteristic of UPEC strains were identified. These included *fimH*, encoding the adhesin subunit of type I fimbriae; the full *pap* operon (*papB–K*, *papX*) responsible for P fimbriae; and the *sfa* gene cluster (*sfaB–Y*) associated with S fimbriae. These structures play key roles in adherence to the uroepithelium, invasion of renal tissues, and establishment of chronic urinary tract infections.

In terms of immune evasion, the capsule biosynthesis and export system (*kpsD*, *kpsM*, *kpsT*) was detected, providing protection against complement-mediated lysis. This system is functionally complemented by *ompA*, an outer membrane protein involved in both adhesion and immunomodulation.

**Figure 2:**
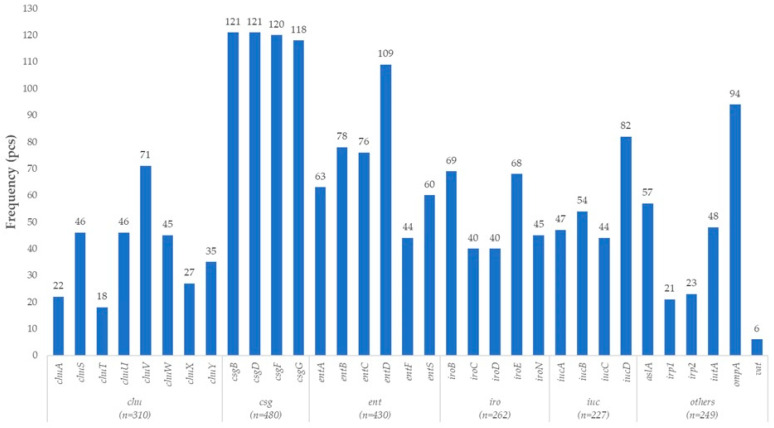
Prevalence of avian pathogenic *Escherichia coli* (APEC)-associated virulence genes in turkey-derived *Escherichia coli* isolates (*n* = 160).

The *E. coli* isolates derived from turkeys exhibited a pronounced gene profile indicative of an APEC phenotype (Figure 2). Colonization and tissue adherence were supported by multiple complementary factors. The *aslA* gene, encoding an outer membrane lipoprotein, promotes adhesion, while *ompA* encodes a well-characterized multifunctional protein involved in both adhesion and immune modulation.

Key components of the curli biosynthesis system (*csgB*, *csgD*, *csgF*, *csgG*), which are essential for biofilm formation and environmental persistence, were also widely present, facilitating surface attachment and long-term survival in the host.

Regarding iron acquisition, a critical aspect of adaptation to the host environment, the isolates exhibited a highly “iron-competent” profile. Genes of the *chu* operon (*chuA*, *chuS*, *chuT*, *chuU*, *chuV*, *chuW*, *chuX*, *chuY*) enable utilization of heme as an iron source. In parallel, the classic enterobactin biosynthesis and export pathways were confirmed by the presence of *entA–F* and *entS*. Additionally, the salmochelin system (*iroB*, *iroC*, *iroD*, *iroE*, *iroN*), the yersiniabactin gene cluster (*irp1*, *irp2*), and the aerobactin pathway (*iucA–D*, *iutA*) were also detected. The co-occurrence of multiple, partially redundant siderophore systems reflects a robust iron acquisition strategy—a hallmark adaptive trait of APEC strains—and has been linked to bacterial proliferation in extraintestinal tissues such as the air sacs, liver, and pericardium.

The virulence repertoire also included the *vat* gene, which encodes a vacuolating autotransporter toxin. This toxin contributes to host cell damage and immune evasion, potentially exacerbating disease severity.

Overall, the detected APEC-associated genes, including adhesion and colonization factors (*aslA*, *ompA*, *curli*), a suite of functionally overlapping iron acquisition systems (*chu*, *ent*, *iro*, *irp*, *iuc/iut*), and the *vat* autotransporter toxin, form a synergistic network of virulence functions. Together, they support effective colonization, nutrient acquisition, and immune evasion in the turkey host. This pattern aligns closely with known APEC pathogenesis mechanisms and suggests that the examined isolates possess a potentially high level of extraintestinal virulence capacity in turkeys.

**Figure 3:**
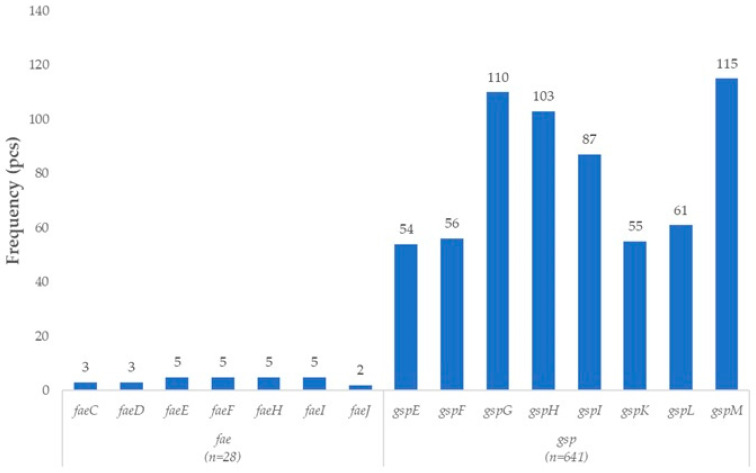
Prevalence of enterotoxigenic *Escherichia coli* (ETEC)-associated virulence genes in turkey-derived *Escherichia coli* isolates (*n* = 160).

The *E. coli* isolates of turkey origin also harbored a gene repertoire characteristic of ETEC pathotypes (Figure 3). Among the most prominent components was the gene cluster responsible for the biosynthesis and assembly of F4 (K88) fimbriae (*faeC*, *faeD*, *faeE*, *faeF*, *faeH*, *faeI*, *faeJ*), which represent the primary colonization factor of ETEC strains. The F4 fimbriae mediate direct adherence to intestinal epithelial cells, playing a critical role in the pathogenesis of diarrheal disease, particularly in young animals.

In parallel, genes encoding components of the type II secretion system (T2SS) were also detected, including *gspE*, *gspF*, *gspG*, *gspH*, *gspI*, *gspK*, *gspL*, and *gspM*. This secretion system enables the export of various extracellular toxins and virulence factors. The function of T2SS is closely linked to enterotoxin secretion and further enhances the enteropathogenic potential of these isolates.

The turkey-derived *E. coli* isolates also harbored several genes associated with virulence mechanisms characteristic of EHEC and EPEC *E. coli* strains. A wide array of type III secretion system (T3SS)-related effector proteins was identified, including *espL1*, *espL4*, *espR1*, *espR3*, *espR4*, *espX1*, *espX2*, *espX4*, *espX6*, and *espY1–Y4*. These genes are primarily associated with the locus of enterocyte effacement (LEE) pathogenicity island, a central virulence determinant in EPEC and EHEC pathogenesis. The effector proteins encoded by the *esp* genes manipulate the host cell cytoskeleton, alter adhesion structures, and modulate host signaling pathways, thereby facilitating intimate bacterial adhesion and promoting the formation of characteristic attaching and effacing (A/E) lesions.

Genes associated with type I fimbriae (*fimA*, *fimB*, *fimC*, *fimD*, *fimE*, *fimF*, *fimG*, *fimI*) were also present. These structures are essential for early-stage colonization and adhesion to host epithelial cells and further contribute to biofilm formation and persistence within the host.

Additional components of the secretion machinery were encoded by genes of the type II secretion system (T2SS), including *gspE*, *gspF*, *gspG*, *gspH*, *gspI*, *gspK*, *gspL*, and *gspM*. This system mediates the export of extracellular toxins and enzymes and is known to be involved in virulence factor secretion in various enteric pathotypes, particularly in ETEC, but also in EPEC and EHEC strains.

**Figure 4:**
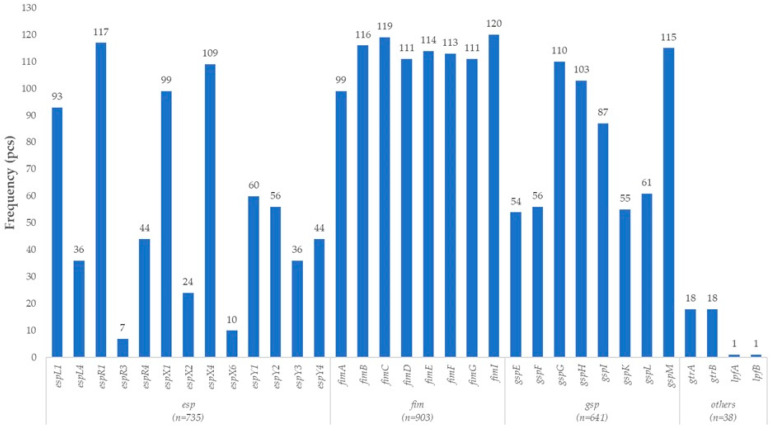
Prevalence of enterohemorrhagic (EHEC) and enteropathogenic (EPEC) *Escherichia coli*-associated virulence genes in turkey-derived *Escherichia coli* isolates (*n* = 160).

Moreover, genes involved in O-antigen modification and variability (*gtrA*, *gtrB*) were detected. These may contribute to immune evasion and increased serotype diversity. The *lpfA* and *lpfB* genes, encoding components of long polar fimbriae (LPF), were also present; these structures are critical for adhesion to intestinal epithelial cells and play a key role in EHEC/EPEC colonization (Figure 4).

Taken together, the gene profiles identified in the turkey-derived *E. coli* isolates reveal a virulence arsenal enriched in LEE-associated type III secretion system (T3SS) effectors, classical fimbrial adhesion factors, type II secretion system (T2SS) components, and long polar fimbriae (LPF) structures. This combination strongly suggests an EPEC/EHEC-like pathogenic potential, supporting efficient host cell colonization, disruption of epithelial integrity, and evasion of host immune responses.

## 4. Discussion

A total of 160 *E. coli* strains isolated from large-scale turkey farms in Hungary were analyzed. These isolates originated from healthy turkeys, including both broiler and breeder flocks. Following phenotypic pre-screening for multidrug resistance and subsequent next-generation sequencing, we characterized the virulence gene content of these strains.

### 4.1. Adhesion-Related Genes

The turkey-derived isolates revealed a partially redundant arsenal of adhesion-associated genes—including curli, various fimbrial systems, and outer membrane proteins—that closely resemble those described in APEC/ExPEC strains with tissue tropism and colonization potential. The presence of curli biosynthesis genes (*csgB*, *csgD*, *csgF*, *csgG*) together with *ompA* constitutes a genetic profile that, according to several studies, promotes host cell adhesion, biofilm formation, and persistence within the avian host. Both original research and systematic reviews have emphasized their functional roles in colonization processes and pathogenicity in birds [13,59].

Among classical chaperone–usher fimbrial systems, type 1 fimbriae (*fimA–I*, especially *fimH*) and P fimbriae (*pap* operon) represent key mediators of early-stage adhesion and tissue specificity [60]. In UPEC, these fimbriae have been experimentally confirmed to facilitate colonization of the uroepithelium and, through synergistic interactions, contribute to infection persistence. Although the presence of these genes does not guarantee their expression, numerous studies have confirmed their functional relevance and complex regulatory roles of the *fim* and *pap* operons [61,62]. Detection of the S fimbriae (*sfa* operon) further supports the high epithelial adhesion potential of the strains, consistent with UPEC-associated findings where S fimbriae promote tropism toward the upper urinary tract [63].

Particular attention is warranted for the *E. coli* common pilus (ECP; *ecp/yag* operon), which was also detected in the turkey isolates. ECP is described as a shared, broad-spectrum adhesion and biofilm factor among multiple pathotypes (EPEC/ExPEC) [64]. In EPEC, it has been shown to synergize with other pili (e.g., BFP) during localized adherence. Thus, the presence of ECP may indicate a multi-modal adhesion strategy, potentially enhancing the pathogenicity of the strains [65,66].

The detection of long polar fimbriae (*lpfA*, *lpfB*) is in line with EHEC/EPEC-like intestinal adherence mechanisms, facilitating binding to enterocytes and contributing to epithelial barrier disruption. This combination may extend enteric colonization capacity in poultry as well [67].

Several adhesion-related genes, such as curli components and fimbriae, are also commonly found in commensal *E. coli*. This underscores the importance of functional validation before attributing pathogenic potential. Future studies should therefore incorporate transcriptomic and phenotypic analyses, such as gene expression profiling, adhesion or biofilm assays to confirm whether these genes are actively expressed and functionally relevant under host-specific conditions. To determine their actual role in virulence, gene expression and activity should be assessed using assays for adhesion, biofilm formation, or cell/tissue interactions in turkey-derived models. Importantly, the expression of these genes is known to be influenced by environmental cues, including temperature, nutrient availability, and host-derived factors. Therefore, genomic data should be complemented by transcriptomic, proteomic, and phenotypic analyses [59].

### 4.2. Iron Acquisition Systemst

A central feature of the fitness-associated genes identified in the turkey-derived isolates is the presence of redundant iron acquisition systems, which support persistence in the iron-limited environment of the host. The catecholate-type siderophore enterobactin (*ent/fep*) is a well-known ExPEC core virulence determinant. However, it is specifically neutralized by host lipocalin-2, a constraint that is circumvented by the salmochelin system (*iroBCDEN*, *iroN*), which produces glycosylated enterobactin capable of evading lipocalin-2 sequestration. This confers a virulence advantage in both enteric and extraintestinal infections [68,69]. The presence of these systems in avian APEC and human ExPEC isolates underscores their epidemiological overlap, especially given that *ColV/ColBM* plasmids—known vectors of virulence—frequently encode iron acquisition genes such as *iuc/iutA* and *iro*, and are epidemiologically associated with higher pathogenic potential [70]. Functional experiments have shown that deletion of the aerobactin system (*iucA–D*, *iutA*) significantly attenuates APEC virulence and reduces competitive fitness in avian models, confirming its critical role in successful in vivo infection [71,72].

The yersiniabactin system (*irp/ybt*, *fyuA*) has emerged as a non-redundant ExPEC virulence factor, particularly in UPEC, where it contributes not only to iron acquisition but also to copper and oxidative stress modulation and metabolic adaptation, thereby enhancing uropathogenicity [73,74]. The diversification of iron sources is further supported by heme utilization pathways. *ChuA*, a TonB-dependent outer membrane receptor, and *Hma* represent independent mechanisms for heme uptake, and are both implicated in colonization and virulence in ExPEC and UPEC strains [75,76].

From the perspective of immune evasion and serum resistance, the group 2 capsule (*kpsD*, *kpsM*, *kpsT*) plays a pivotal role. K1/K2 capsule types are well-established ExPEC virulence markers, enhancing resistance to complement-mediated killing and contributing to the severity of bacteremia and urinary tract infections. More recent studies have highlighted the synergistic interaction between capsule and LPS structures in conferring serum resistance [77,78]. In line with this, the *waaF* and *waaG* glycosyltransferase genes involved in LPS core biosynthesis are key structural elements. Mutations at these loci increase outer membrane permeability and susceptibility to antimicrobial peptides, underscoring the protective role of core oligosaccharides in fitness and immune evasion [79].

Genes involved in flagellar structure and motility (e.g., *flg*, *fli*, *flh*, *mot*) facilitate adaptation to diverse environmental and host tissue niches. In UPEC models, flagellum-mediated swimming and chemotaxis have been shown to confer a competitive advantage in colonization, tissue dissemination, and immune evasion. Expression of motility genes coincides temporally with key phases of infection and facilitates ascending spread in the urinary tract. Although our study focused on turkey-derived isolates, the adaptive value of motility may extend to persistence in both intestinal and respiratory niches of poultry [80].

### 4.3. Secretion and Toxin Genes

The LEE-associated type III secretion system (T3SS) effectors identified in the isolates—particularly members of the *espL*, *espR*, *espX*, and *espY* families—point toward a pathogenic mechanism central to EPEC/EHEC virulence. The LEE island encodes the intimin-based intimate adherence system and the T3SS translocon, which collectively mediate actin pedestal formation and remodeling of epithelial junctions, leading to attaching and effacing (A/E) lesions. These mechanisms and the functional diversity of *esp* effectors are well documented in EPEC/EHEC models and strongly correlate with epithelial barrier disruption and sustained colonization [81,82]. Genomic studies of EHEC O157:H7 have revealed a high number and diversity of T3SS effectors, suggesting potential for functional redundancy and synergy [83].

The type II secretion system (T2SS, *gspE–M*) is also considered pathophysiologically relevant across multiple pathotypes. In ETEC, the T2SS is the principal secretion pathway for heat-labile enterotoxin (LT), and T2SS ATPase activity (*gspE*) is directly associated with LT export and virulence phenotype [84]. In EHEC and EPEC backgrounds, the T2SS facilitates the export of non-enterotoxin substrates as well. For example, the O157-encoded T2SS on the pO157 plasmid mediates secretion of the StcE metalloprotease and the YodA/ZinT proteins, which modulate host responses by targeting mucus layers and complement regulators [85].

Among the autotransporter toxins (SPATEs) detected, Pic and Vat deserve particular attention. *Pic* is a serine protease autotransporter with mucolytic activity that alters the mucus layer, facilitating access to the intestinal epithelium and exerting immunomodulatory effects. Its role has been demonstrated in EAEC and other pathotypes [86]. *Vat* (vacuolating autotransporter toxin) is a class II SPATE identified in APEC/ExPEC strains, with cytotoxic and vacuolating activity. Notably, its biological activity has been confirmed in avian cell systems (e.g., chicken embryonic fibroblasts), which is directly relevant in the context of the turkey host [87]. Comprehensive APEC studies highlight that *vat* often co-occurs with other ExPEC-associated factors such as *chuA*, *fyuA*, *irp2*, *iutA*, *iroN*, and *ompT*—a gene constellation linked to extraintestinal behavior and zoonotic potential [88].

These findings outline a multilayered virulence architecture, where LEE-based T3SS effectors disrupt epithelial function and mediate intimate adherence, the T2SS secretes enterotoxins and host-modulating proteins, and SPATE autotransporters act to “soften” the mucus and mucosal defenses, thereby facilitating colonization.

The pronounced presence and functional relevance of *vat* within an APEC-compatible genetic background is especially important in assessing health risks for turkey flocks and aligns with international findings. Moreover, the UPEC-like virulence gene repertoire found in turkey isolates is strikingly congruent with major determinants of human UPEC, including the high prevalence of *fimH*, *pap*, and *sfa*, as well as the presence of multiple siderophore systems—key to uropathogenic tropism and persistence.

Functional studies have shown that deletion of the aerobactin system (*iucA–D*, *iutA*) significantly impairs ExPEC/UPEC virulence, whereas loss of other siderophores (enterobactin, salmochelin, yersiniabactin) has a more limited effect. Hem receptors (*chuA*, *hma*) further contribute to renal colonization, while capsule expression—especially K1/K2 types—enhances complement evasion and serum resistance. Taken together, these data suggest that the UPEC-compatible genotype of the turkey-derived isolates may confer genuine pathogenic potential, particularly if linked to ColV/ColBM-type plasmids [89].

The APEC gene repertoire detected is consistent with the biological basis of colibacillosis in poultry, including tropism for the air sacs, liver, and pericardium. Reviews on APEC virulence and zoonotic potential emphasize the central role of “core” genes encoded on ColV-type plasmids—*iuc/iutA*, *iroN*, *iss*, and *hlyF*—which are highly prevalent in isolates from diseased poultry. The cytotoxic activity of *vat* has also been experimentally demonstrated in chicken embryonic fibroblasts, supporting its role in tissue damage in both APEC and ExPEC contexts. Epidemiological data further point to overlaps between human ExPEC and APEC lineages in terms of sequence types (STs), serogroups, and virulence-associated gene (VAG) patterns, strengthening the case for zoonotic transmission potential [87,88].

The presence of LEE-associated T3SS effectors (*espL*, *espR*, *espX*, *espY* families) points to an EPEC/EHEC-like attaching and effacing (A/E) pathogenesis model, where intimin-mediated intimate adherence and actin cytoskeleton remodeling are key to epithelial disruption and sustained colonization. The regulatory network controlling LEE expression and the functional diversity of effectors is well characterized. Additionally, long polar fimbriae (*lpfA*, *lpfB*) have been shown in EHEC to mediate adherence to intestinal epithelium and interaction with Peyer’s patches. The ECP (*ecp/yag* operon), shared among multiple pathotypes, facilitates intestinal colonization and biofilm formation. The simultaneous presence of LPF, ECP, and LEE elements in the isolates suggests a robust adhesion and persistence capacity in the intestinal niche [82,90].

The F4 (K88) fimbrial biosynthesis genes (*faeC–J*) represent classic colonization determinants in enterotoxigenic *E. coli* (ETEC), mediating adhesion to enterocytes, particularly in neonatal and young animals. The concurrent detection of the type II secretion system (T2SS; *gspE–M*) is pathogenetically relevant, as T2SS is required for the secretion of heat-labile enterotoxin (LT) in ETEC. Experimental data demonstrates a correlation between GspE ATPase activity and LT secretion levels. Together, the presence of F4 fimbriae and T2SS-mediated toxin export suggests a potential for enteric pathogenicity that may hold clinical relevance in poultry as well [91].

Although *pic* is primarily associated with EAEC, its presence across multiple pathotypes highlights its broader functional significance. With mucolytic and complement-modulatory activity, *pic* contributes to mucus remodeling and immune evasion. In conjunction with LEE and T2SS systems, this supports the emerging “hetero-/hybrid pathotype” concept, in which *E. coli* strains harbor virulence factors from multiple pathogroups [86].

Gene presence alone does not guarantee functional expression. To assess pathogenic relevance, targeted phenotypic assays—such as adhesion and biofilm formation, toxin secretion, and iron acquisition competition—are needed. Ideally, these should be performed using turkey-derived cell lines or in vivo poultry models [88].

The virulence gene content of turkey-derived isolates displays a mosaic, multilayered organization. This includes overlapping colonization systems (e.g., fimbriae, curli, ECP), redundant iron acquisition strategies (e.g., enterobactin, salmochelin, aerobactin, yersiniabactin, heme uptake), and a variety of secretion mechanisms and effectors (e.g., T3SS/LEE, T2SS, SPATEs). This genomic configuration likely confers a selective advantage across both intestinal and extraintestinal environments. Similar patterns have been observed globally: the LEE-encoded T3SS mediates intimate adherence and A/E lesion formation in EPEC and EHEC; the T2SS facilitates LT secretion, a hallmark of enteric disease; and salmochelin bypasses host lipocalin-2 sequestration, thereby restoring enterobactin-dependent iron uptake and enhancing bacterial fitness [92,93].

From an APEC-focused perspective, the co-occurrence of ColV-associated iron acquisition modules (*iuc/iutA*, *iro*), ExPEC-compatible capsule/LPS shields, and SPATE autotransporters (*vat*, *pic*) defines a core virulence backbone that has been linked to zoonotic transmissibility in multiple independent comparative and epidemiological studies. This concern is further heightened by the concurrent presence of resistance determinants (e.g., ESBLs), whose spread within the poultry production chain has been well documented, reinforcing the One Health relevance of these findings and the urgent need for targeted, host-specific interventions [94].

Still, genomic presence alone does not imply functional virulence. Transcriptomic, proteomic, and phenotypic validation is essential for causal interpretation. Our findings therefore provide genomic evidence for a high-risk, host-adapted, and potentially hybridizing virulence repertoire in turkeys. The next critical steps are functional verification and the development of integrated precision control strategies, including farm-level biosecurity and antimicrobial stewardship. These actions promise mutual benefits for poultry health and food chain safety alike, reflecting the interdependence at the core of One Health frameworks [95].

## 5. Conclusions

This study provides the first comprehensive whole-genome-based characterization of ESBL-producing *E. coli* isolates from intensively reared turkeys in Hungary. Against the backdrop of growing concerns around antimicrobial resistance and zoonotic transmission, our objective was to map the virulence gene repertoire and assess the potential public health relevance of these avian isolates within the One Health framework.

Whole-genome analyses revealed a complex and host-adapted virulence architecture, including colonization-associated modules (type I, P, and S fimbriae, ECP, curli), multiple iron acquisition systems (enterobactin, salmochelin, aerobactin, yersiniabactin, and heme utilization), and functionally diverse secretion pathways (LEE-associated T3SS and T2SS). Notably, genetic hallmarks of APEC-, UPEC-, and EPEC/EHEC pathotypes were co-localized within single isolates, often in conjunction with ESBL markers and plasmid elements (e.g., ColV-like plasmids), suggesting the existence of mosaic genomes with enhanced ecological fitness and zoonotic potential.

These findings highlight a previously underappreciated reservoir of virulence and resistance genes in poultry-associated *E. coli*, with significant implications for food safety and public health. Importantly, the study addresses a gap in our understanding of the genomic convergence of ExPEC and diarrheagenic traits in avian isolates. However, the pathogenic relevance of several identified factors requires further validation in turkey-specific experimental models.

Moving forward, integrated strategies are needed at multiple levels. In the short term, routine PCR-based and WGS-based monitoring of key virulence and ESBL markers is warranted in poultry farms. In the medium term, functional assays, including adhesion, biofilm formation, siderophore competition, and secretion system activity, should be employed to link genomic content to phenotypic expression. In the long term, rational development of virulence-interference strategies targeting conserved elements such as aerobactin/salmochelin systems and SPATE autotransporters may offer novel avenues for disease mitigation.

Overall, this work establishes a genomic and functional foundation for targeted surveillance and intervention in turkey production. By demonstrating the potential zoonotic threat of ESBL-producing *E. coli* with hybrid pathotype features, the study underscores the urgency of implementing genomic surveillance programs in poultry farms as a cornerstone of antimicrobial stewardship and One Health preparedness.

## Figures and Tables

**Table 1:** Presence of colonization-associated virulence genes (e.g., fimbriae, curli, ECP) detected in whole-genome sequences of *Escherichia coli* isolates from domestic turkeys. Gene functions and pathotype associations (APEC, UPEC, EPEC) are indicated where applicable.

Class	VFG (%)	Function	Pathotype
Colonization	*aslA* (14%)	Outer membrane protein promoting adhesion	APEC
	*csgB* (25%)	Curli fiber subunit, involved in adhesion and biofilm formation	APEC, UPEC
	*csgF* (25%)	Curli secretion and assembly	
	*csgG* (25%)	Outer membrane protein involved in curli secretion	
	*faeC* (11%)	Adhesion in F4 fimbriae biosynthesis	ETEC
	*faeD* (11%)	Assembly of F4 fimbriae	
	*faeE* (18%)	Transport of fimbrial subunits	
	*faeF* (18%)	Anchoring of F4 fimbriae	
	*faeH* (18%)	Fimbrial biogenesis	
	*faeI* (18%)		
	*faeJ* (7%)		
	*fdeC* (7%)	Fibronectin adhesin, epithelial binding	UPEC
	*fimA* (7%)	Type I fimbriae major subunit	EPEC, EHEC
	*fimB* (36%)	Fimbrial phase variation control	
	*fimC* (36%)	Chaperone in fimbrial assembly	
	*fimD* (47%)	Fimbrial usher protein	
	*fimE* (29%)	Fimbrial expression regulator	
	*fimF* (29%)	Fimbrial assembly	
	*fimG* (37%)		
	*fimH* (29%)	Fimbrial adhesin	UPEC
	*fimI* (46%)	Minor fimbrial component	EPEC, EHEC
	*focH* (14%)	F1C fimbrial adhesin	UPEC
	*ibeA* (14%)	Invasion of brain endothelium protein	MNEC
	*lpfA* (14%)	Long polar fimbriae subunit	EHEC, EPEC
	*lpfB* (8%)	Long polar fimbriae assembly protein	
	*ompA* (24%)	Outer membrane protein A, adhesion	UPEC, APEC
	*papB* (2%)	Regulatory protein for P fimbriae	UPEC
	*papD* (2%)	P fimbrial chaperone	
	*papE* (2%)	P fimbrial minor subunit	
	*papF* (2%)	P fimbrial minor subunit	
	*papG* (2%)	P fimbrial adhesin	
	*papH* (2%)	Fimbrial anchoring subunit	
	*papI* (2%)	Transcriptional regulator of pap operon	
	*papK* (2%)	P fimbriae, structural subunit	
	*papX* (2%)	P fimbriae regulatory protein	
	*pilG* (6%)	Type IV pilus biogenesis protein	EPEC
	*pilH* (6%)	Type IV pilus assembly ATPase	
	*sfaB* (2%)	S fimbriae assembly protein	UPEC
	*sfaC* (2%)	S fimbriae chaperone	
	*sfaD* (2%)	S fimbriae minor subunit	
	*sfaE* (2%)	S fimbriae structural subunit	
	*sfaF* (2%)	S fimbriae usher protein	
	*sfaG* (2%)	S fimbriae structural component	
	*sfaX* (2%)	S fimbriae regulatory protein	
	*sfaY* (2%)	S fimbriae expression modulator	
	*yagV/ecpE* (16%)	*E. coli* common pilus structural protein	EPEC
	*yagW/ecpD* (18%)	*E. coli* common pilus usher	
	*yagX/ecpC* (18%)	*E. coli* common pilus chaperone	
	*yagY/ecpB* (18%)	*E. coli* common pilus minor subunit	
	*yagZ/ecpA* (16%)	*E. coli* common pilus major structural subunit	
	*ykgK/ecpR* (16%)	Regulator of *ecp* operon (*E. coli* common pilus)	

VFG—Virulence factor gene, UPEC—uropathogenic *Escherichia coli*, EPEC—enteropathogenic *Escherichia coli*, APEC—avian pathogenic *Escherichia coli*, ETEC—enterotoxigenic *Escherichia coli*, MNEC—meningitis-associated *Escherichia coli.*

**Table 2:** Fitness-associated virulence genes identified in *Escherichia coli* pathotypes originating from turkeys.

Class	VFG	Function	Pathotype
Fitness	*alg8* (14%)	Alginate biosynthesis protein, contributes to biofilm formation	UPEC
	*algA* (14%)	Involved in the synthesis of GDP-mannose for alginate production	
	*algB* (14%)	Regulatory protein involved in alginate biosynthesis	
	*algC* (14%)	Phosphomannomutase/phosphoglucomutase for polysaccharide biosynthesis	
	*algI* (14%)	Involved in O-acetylation of alginate	
	*algU* (14%)	Sigma factor controlling alginate biosynthesis	
	*algW* (14%)	Protease involved in stress response and alginate production	
	*chuA* (7%)	Outer membrane heme receptor for iron uptake	UPEC, APEC
	*chuS* (14%)	Heme degradation protein for iron acquisition	
	*chuT* (6%)	Periplasmic heme-binding protein	
	*chuU* (19%)	ABC transporter permease protein for heme	
	*chuV* (22%)	ABC transporter ATP-binding protein	
	*chuW* (14%)	Associated with heme utilization	
	*chuX* (8%)	Putative heme transport protein	
	*chuY* (11%)	Ferric iron reductase	
	*entA* (13%)	Siderophore (enterobactin) biosynthesis	
	*entB* (16%)	Siderophore biosynthesis	
	*entC* (16%)		
	*entD* (22%)		
	*entF* (12%)		
	*entS* (9%)	Enterobactin exporter protein	
	*fepA* (5%)	Ferric enterobactin receptor	ExPEC
	*fepB* (5%)	Periplasmic binding protein for ferric enterobactin	
	*fepC* (5%)	ABC transporter ATP-binding protein	
	*fepD* (5%)	Transport system permease protein	
	*fepG* (5%)	Component of enterobactin transport system	
	*fes* (5%)	Enterobactin esterase, releases iron	
	*fleN* (7%)	Flagellar biosynthesis regulation	
	*fleQ* (7%)	Master regulator of flagella	
	*flgC* (7%)	Basal body rod component	
	*flgG* (7%)		
	*flgH* (7%)	L ring protein of flagella	
	*flgI* (7%)	P ring protein of flagella	
	*flhA* (7%)	Flagellar export apparatus protein	
	*fliA* (7%)	Sigma factor for flagellar operon	
	*fliG* (7%)	Motor switch complex protein	
	*fliI* (7%)	Flagellar ATPase	
	*fliM* (7%)	Flagellar motor switch	
	*fliN* (5%)	Motor switch complex protein	
	*fliP* (5%)	Export apparatus membrane protein	
	*fyuA* (4%)	Yersiniabactin receptor	UPEC
	*gtrA* (6%)	Glycosyltransferase-associated protein involved in O-antigen modification	EPEC, EHEC
	*gtrB* (6%)	Glycosyltransferase involved in antigen variation	
	*iroB* (26%)	Glycosyltransferase for salmochelin siderophore	UPEC, APEC
	*iroC* (15%)	Salmochelin exporter	
	*iroD* (15%)	Salmochelin esterase	
	*iroE* (26%)	Periplasmic esterase for salmochelin	
	*iroN* (17%)	Outer membrane receptor for salmochelin	
	*irp1* (8%)	Siderophore biosynthesis (yersiniabactin)	
	*irp2* (9%)		
	*iucA* (21%)	Aerobactin biosynthesis	
	*iucB* (24%)		
	*iucC* (19%)		
	*iucD* (36%)		
	*iutA* (19%)	Aerobactin receptor	
	*kpsD* (38%)	Capsule export protein	UPEC, MNEC
	*kpsM* (48%)	Capsule export inner membrane protein	
	*kpsT* (14%)	Capsule export ATP-binding protein	
	*mbtH-like* (5%)	Involved in siderophore biosynthesis	ExPEC
	*motB* (5%)	Flagellar motor protein	
	*motC* (5%)	Putative flagellar-related protein	
	*mucD* (5%)	Serine protease involved in stress response and possibly virulence regulation	
	*pvdH* (5%)	Involved in pyoverdine biosynthesis	
	*pvdS* (5%)	Sigma factor for pyoverdine synthesis	
	*shuA* (6%)	Heme receptor	
	*shuS* (6%)	Involved in heme utilization	
	*shuT* (6%)	ABC transporter substrate-binding protein	
	*shuV* (6%)	ABC transporter permease	
	*shuX* (6%)	ABC transporter ATP-binding protein	
	*shuY* (6%)	ABC transporter-related, heme utilization	
	*waaF* (2%)	LPS core heptosyltransferase II	
	*waaG* (2%)	LPS core glucosyltransferase I	
	*ybtA* (2%)	Transcriptional regulator of yersiniabactin system	UPEC
	*ybtE* (2%)	Yersiniabactin biosynthesis	
	*ybtP* (2%)	Yersiniabactin ABC transporter permease	
	*ybtQ* (2%)	Yersiniabactin ABC transporter ATP-binding	
	*ybtS* (2%)	Yersiniabactin biosynthesis	
	*ybtT* (2%)		
	*ybtU* (2%)		
	*ybtX* (2%)	Yersiniabactin exporter	

VFG—Virulence factor gene, UPEC—uropathogenic *Escherichia coli*, APEC—avian pathogenic *Escherichia coli*, EHEC—enterohemorrhagic *Escherichia coli*, ExPEC—extraintestinal pathogenic *Escherichia coli*, MNEC—meningitis-associated *Escherichia coli.*

**Table 3:** Toxin- and effector-associated virulence genes identified in *Escherichia coli* pathotypes originating from turkeys.

Class	VFG	Function	Pathotype
Toxins	*pic* (2%)	Serine protease autotransporter, involved in immune evasion	EAEC
	*vat* (2%)	Vacuolating autotransporter toxin	APEC
Effectors	*espL1* (13%)	Type III secretion system effector protein	EHEC, EPEC
	*espL4* (5%)	T3SS effector, role in host interaction	
	*espR1* (16%)	Regulator of LEE operon expression	
	*espR3* (1%)	Regulatory role in secretion system	
	*espR4* (6%)	Regulatory protein related to LEE	
	*espX1* (13%)	T3SS effector with unknown specific role	
	*espX2* (3%)	T3SS effector protein	
	*espX4* (15%)		
	*espX6* (1%)		
	*espY1* (8%)	T3SS effector protein affecting cytoskeleton	
	*espY2* (8%)	T3SS effector	
	*espY3* (5%)		
	*espY4* (6%)		
	*gspC* (8%)	Type II secretion system component	ExPEC
	*gspD* (9%)	Outer membrane secretin of T2SS	
	*gspE* (17%)	Type II secretion system protein	EHEC, ETEC
	*gspF* (16%)		
	*gspG* (14%)	Type II secretion system pseudopilin	
	*gspH* (9%)		
	*gspI* (10%)		
	*gspK* (10%)	Type II secretion system protein	
	*gspL* (10%)		
	*gspM* (18%)		

VFG—Virulence factor gene, APEC—avian pathogenic *Escherichia coli*, EHEC—enterohemorrhagic *Escherichia coli*, ETEC—enterotoxigenic *Escherichia coli,* EAEC—enteroaggregative *Escherichia coli*, ExPEC—extraintestinal pathogenic *Escherichia coli.*

## Data Availability

The original contributions presented in this study are included in the article/Supplementary Material. Further inquiries can be directed to the corresponding author.

## Supplementary Materials

The following supporting information can be downloaded at: https://www.mdpi.com/article/10.3390/vetsci12121141/s1, Additional excel (turkeys).
